# Relationship Between Resting State Functional Connectivity and Reading-Related Behavioural Measures in 69 Adults

**DOI:** 10.1162/nol_a_00146

**Published:** 2024-06-14

**Authors:** Joe Bathelt, Kathleen Rastle, J. S. H. Taylor

**Affiliations:** Department of Psychology, Royal Holloway University of London, UK; Faculty of Social and Behavioural Sciences, University of Amsterdam, Netherlands; Division of Psychology and Language Sciences, University College London, London, UK

**Keywords:** fMRI, lexical, phonological, reading, resting state functional connectivity, visual word form area

## Abstract

In computational models of reading, written words can be read using print-to-sound and/or print-to-meaning pathways. Neuroimaging data associate dorsal stream regions (left posterior occipitotemporal cortex, intraparietal cortex, dorsal inferior frontal gyrus [dIFG]) with the print-to-sound pathway and ventral stream regions (left anterior fusiform gyrus, middle temporal gyrus) with the print-to-meaning pathway. In 69 typical adults, we investigated whether resting state functional connectivity (RSFC) between the visual word form area (VWFA) and dorsal and ventral regions correlated with phonological (nonword reading, nonword repetition, spoonerisms), lexical-semantic (vocabulary, sensitivity to morpheme units in reading), and general literacy (word reading, spelling) skills. VWFA activity was temporally correlated with activity in both dorsal and ventral reading regions. In pre-registered whole-brain analyses, spoonerisms performance was positively correlated with RSFC between the VWFA and left dorsal regions (dIFG, superior parietal and intraparietal cortex). In exploratory region-of-interest analyses, VWFA-dIFG connectivity was also positively correlated with nonword repetition, spelling, and vocabulary. Connectivity between the VWFA and ventral stream regions was not associated with performance on any behavioural measure, either in whole-brain or region-of-interest analyses. Our results suggest that tasks such as spoonerisms and spellings, which are both complex (i.e., involve multiple subprocesses) and have high between-subject variability, provide greater opportunity for observing resting-state brain-behaviour associations. However, the complexity of these tasks limits the conclusions we can draw about the specific mechanisms that drive these associations. Future research would benefit from constructing latent variables from multiple tasks tapping the same reading subprocess.

## INTRODUCTION

**Figure 1.**
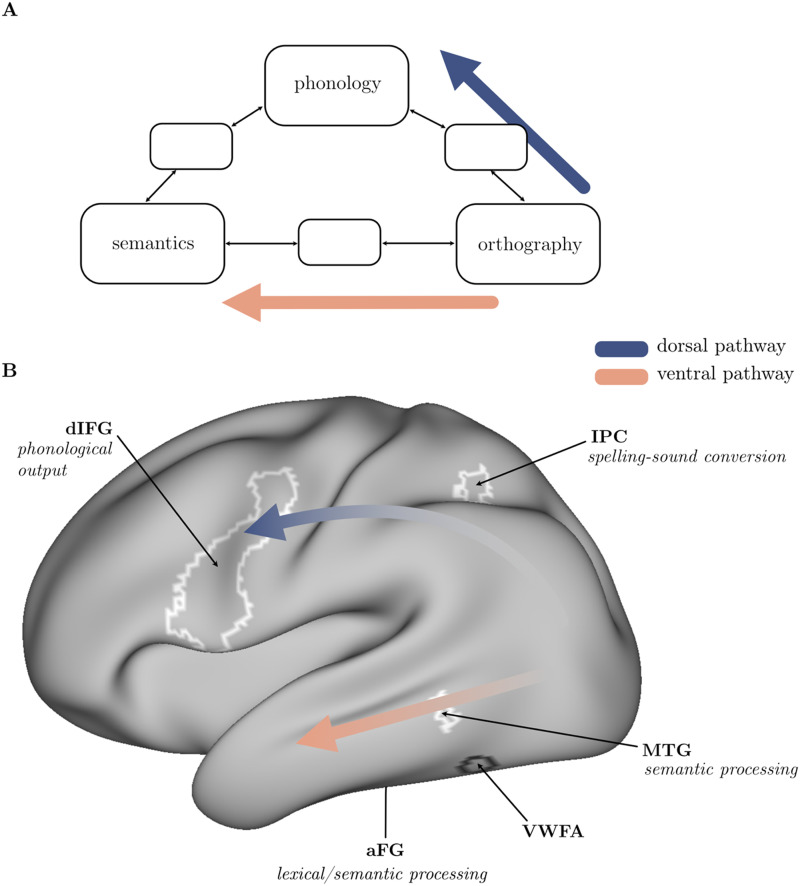
Correspondence between cognitive components of reading and brain areas active during reading, adapted from Taylor et al. (2013). The blue arrows illustrate the print-to-sound (dorsal) pathway and the salmon arrows illustrate the print-to-meaning (ventral) pathway. A. The triangle model of reading (Harm & Seidenberg, 2004; Plaut et al., 1996). B. Brain areas active during reading in Taylor et al. (2013) and their suggested cognitive processes. The faded parts of the arrows indicate overlap between ventral and dorsal pathways in the posterior part of the brain, which is engaged in orthographic processing. *Abbreviations*: dIFG = dorsal inferior frontal gyrus, IPC = intraparietal cortex, MTG = medial temporal gyrus, VWFA = visual word form area, aFG = anterior fusiform gyrus.

Computational models of reading propose that understanding written words can be accomplished via two reading pathways, one that maps from print-to-sound (orthography-to-phonology) and then from sound-to-meaning (phonology-to-semantics), and another that maps directly from print-to-meaning (orthography-to-semantics; Coltheart et al., 2001; Harm & Seidenberg, 2004; Plaut et al., 1996). The print-to-sound pathway supports the phonic decoding processes necessary to read unfamiliar words in alphabetic languages, whereas the print-to-meaning pathway supports whole-word recognition and efficient comprehension. These pathways have been associated with different brain regions (Figure 1). Dorsal stream regions, including left posterior occipitotemporal cortex, intraparietal cortex (IPC), and dorsal inferior frontal gyrus (dIFG), are involved in mapping from print-to-sound. Ventral stream regions, including left mid to anterior occipitotemporal cortex, anterior fusiform gyrus (aFG), and middle temporal gyrus (MTG), as well as angular gyrus, are involved in mapping print-to-meaning (Carreiras et al., 2014; Price, 2012; Taylor et al., 2013). Diffusion tensor imaging has shown that the major white matter (WM) tracts that underpin these pathways include the fronto-temporal and fronto-parietal segments of the arcuate fasciculus and the inferior longitudinal fasciculus (e.g., see Figure 1 in Yablonski et al., 2019).

One promising way to investigate the neural underpinnings of reading skill involves examination of functional networks while participants are at rest. Resting state analyses measure temporal correlations between the blood oxygen level dependent (BOLD) signal of different brain areas. Patterns of resting state functional connectivity (RSFC) are thought to reflect the brain's functional networks (Fox & Raichle, 2007). Investigations of RSFC offer the opportunity to probe these networks in a manner that is uncontaminated by strategies or processes associated with a specific task. This approach is useful for studying reading for several reasons. Firstly, it overcomes the limitations associated with task-based fMRI, such as performance and strategy variability and the complexities of designing tasks that accurately capture the cognitive processes involved in reading. By observing the brain in a resting state, baseline levels of connectivity between the visual word form area (VWFA) and other regions can be identified, providing insights into the fundamental neural networks that underpin reading.

One of the first studies to investigate RSFC between reading-related regions (Vogel et al., 2012) focused on a specific seed region within left FG, namely the VWFA (Dehaene & Cohen, 2011), in 25 children and 23 adults. Surprisingly, they reported that VWFA activity at rest was not temporally correlated with activity in regions proposed to be important for either phonological (left IFG, left supramarginal gyrus [SMG]) or lexical-semantic (left inferior temporal gyrus, left angular gyrus) aspects of reading. There were, however, strong temporal correlations between VWFA activity and activity in the dorsal attention network, namely, bilateral IPC and frontal eye-fields, and the strength of these correlations related to reading ability. Similarly, López-Barroso et al. (2020) identified stronger functional connectivity of the VWFA and the left dorsal attention network in literate compared to illerate participants. Strong resting-state connections between the VWFA and the dorsal attention network were also reported by Ellenblum et al. (2019). Vogel et al. argued that these relationships reflect the high demands placed on the spatial allocation of attention during reading. Others, however, have proposed that left IPC plays a role in sequential spelling-sound conversion during reading (Taylor et al., 2013, 2014).

Other studies have found that the VWFA shows strong RSFC with reading related-regions and that this relates to reading ability. In 25 native English-speaking adults, Koyama et al. (2010) observed significant RSFC between a seed in the left FG and left dIFG and IPC (specifically SMG) as well as left anterior MTG. Koyama et al. (2011) further demonstrated that the strength of the connectivity between left FG and left IFG and IPC was positively correlated with word reading ability in these adults. They also found that, in both children (*n* = 25) and adults, word reading ability was positively correlated with RSFC between left dorsal IFG/posteror central gyrus (PCG) and left posterior superior temporal gyrus (STG) and IPC. Similarly, Stevens et al. (2017) found significant RSFC between left VWFA and left dIFG and PCG and posterior STG in 33 native English-speaking adults. Word reading and picture naming accuracy correlated with the strength of the connection between left VWFA and STG. Cheema et al. (2021) observed significant RSFC between a left FG seed and left intraparietal sulcus, SMG, and STG in 19 English speaking adults. However, connecitivity with left FG did not correlate with word or pseudoword reading. In 42 Chinese adults, Zhang et al. (2014) found that both Chinese and English reading ability was positively correlated with RSFC between left FG and left STG, SMG, and PCG. Overall these studies relatively consistently demonstrate that reading ability is positively related to RSFC between the left FG, implicated in orthographic processing, and left dorsal stream regions, implicated in phonological representation/processing (STG, IPC, SMG) and output (IFG and PCG).

These studies only measured single word and pseudoword reading ability. However, a large study with 8- to 14-year-old children (*N* = 83) examined the relationship between RSFC and several different reading and related measures, including word and pseudoword reading efficiency, reading comprehension, and rapid automatised letter naming (Cross et al., 2021). In analyses controlling for nonverbal ability and age, word reading efficiency positively correlated with RSFC between left thalamus and left FG, whereas reading comprehension correlated with RSFC between left thalamus and right FG and between right superior parietal lobe and left superior/middle frontal gyrus. Pseudoword reading ability did not correlate with RSFC between any regions. However, in analyses that controlled for all other behavioural measures, positive relationships were obtained between pseudoword reading and RSFC between left PCG and right angular and suprmarginal gyri, as well as middle frontal gyrus. Several negative relationships between RSFC and behavioural measures were also observed. This study had a larger sample size than those reported earlier and examined a greater number of reading skills. However, the results are less consistent both with previous studies and with our knowledge of reading related regions from other methods (Carreiras et al., 2014; Price, 2012; Taylor et al., 2013). This indicates the need for further large studies with both children and adults that examine more than just single word reading ability.

The current study therefore measured RSFC in a large sample of typical adult readers and assessed multiple reading and reading related skills that we assumed to tap different cognitive components. We examined correlations between performance on these tasks and RSFC between a seed in VWFA and the rest of the brain. Our preregistered hypotheses were as follows:

**Hypothesis 1**. Performance on phonological tasks, including nonword reading, nonword repetition, and spoonerisms, will be positively correlated with RSFC connectivity between the VWFA seed and dorsal regions, specifically left IPC and dIFG.
**Hypothesis 2**. Performance on lexical-semantic tasks, including vocabulary and sensitivity to morpheme units in reading, will be positively correlated with connectivity between the VWFA seed and ventral regions, specifically, anterior FG and MTG.
**Hypothesis 3**. Performance on whole-word reading and spelling-to-dictation tasks, which tap general reading/spelling skills, will be positively correlated with connectivity between the VWFA seed and both dorsal and ventral regions, specifically, IPC, dIFG, aFG, and MTG.

In considering these hypotheses, it is important to remember that the print-to-sound and print-to-meaning pathways are not independent. Specifically, theories of reading acquisition suggest that good print-to-sound decoders are better equipped to self-teach and develop the print-to-meaning pathway (Pritchard et al., 2018; Share, 1995). This means that, for example, although pseudoword reading depends primarily on processes supported by the dorsal pathway, pseudoword reading skill may correlate with connectivity between left VWFA and both dorsal and ventral pathway regions. Likewise, the tasks we used to probe reading skill do not necessarily isolate very specific processes. Our hypotheses are, therefore, somewhat tentative and we regard our analyses as exploratory and requiring replication. However, our study contributes to the literature by examining the relationship between RSFC and multiple reading/language measures that are widely used in studies with both developing and skilled readers, the dataset is relatively large, and we preregistered our hypotheses and analyses.

## MATERIALS AND METHOD

### Participants

Ethical approval was granted by the research ethics committee at Royal Holloway, University of London. Participants were recruited via local advertising and most were university students. Participants were eligible if they were right-handed and if they did not have any current, or history of, language or reading problems or any other learning disabilities, hearing impairments, or uncorrected vision impairments. Further, participants with counterindications for magnetic resonance imaging (MRI) were excluded (i.e., claustrophobia, any trauma or surgery which may have left magnetic material in the body, presence of magnetic implants or pacemakers). Seventy-one right-handed adults participated. Data were collected as part of three separate projects in November 2014–March 2015, December 2015–February 2016, and January–February 2017. Participants with high movement during the resting-state sequence were excluded (*N* = 2), according to the definition from Power et al. (2012; i.e., frame-wise displacement over 0.5 mm) and DVARS above 5%. Image quality metrics were calculated using MRIQC (Esteban et al., 2017). This resulted in a final sample of 69 participants, which is larger than sample sizes of previous studies with adults that employed similar methods, for example, *N* = 33 (Stevens et al., 2017), *N* = 34 (Cheema et al., 2021), *N* = 25 adults (Koyama et al., 2010; Koyama et al., 2011). The demographics of this final sample were as follows: age (Mean = 20.68; *SD* = 2.70, min = 18, max = 34, 1 participant not included in these values since age was recorded incorrectly), gender (53 females, 16 males).

### Behavioural Measures

Participants undertook a series of tasks to assess a variety of reading and language skills. Tasks were completed in a single session in the order listed. Some additional tasks were also completed but were not included in preregistered analyses. Descriptions and data for all tasks are provided on the open science framework (https://osf.io/yhf2e/).

- ***Sight Word Efficiency subtest from the Test of Word Reading Efficiency (TOWRE-2; Torgersen et al., 2012)*.** Participants read aloud as many as possible of a list of 108 words in 45 s. Items are organised in increasing difficulty. Participants were recorded and scored offline and only correctly pronounced words were included in the total score. These raw scores were used for all analyses.
- ***Phonemic Decoding Efficiency subtest from the Test of Word Reading Efficiency, second edition (TOWRE-2; Torgersen et al., 2012)*.** Participants read aloud as many as possible of a list of 66 pseudowords in 45 s. Items are organised in increasing difficulty. Participants were recorded and scored offline and only plausibly pronounced pseudowords were included in the total score. These raw scores were used for all analyses.
- ***Pseudomorpheme lexical decision task (stimuli from Experiment 1 of Crepaldi et al., 2010)*.** Participants completed a 240 item lexical decision task consisting of 60 suffixed pseudowords (e.g., towerly), 60 pairwise-matched control pseudowords with a non-suffixed ending (e.g., towerla), and 120 real words. Each trial started with a 500 ms fixation cross, followed by the target item, which remained on screen until a response was given, or until a timeout of 2,500 ms. Participants responded using a button box, pressing the right button for words and the left button for pseudowords. The measure used for analyses was the pseudomorpheme accuracy cost, calculated as the mean accuracy for control pseudowords minus the mean accuracy for suffixed pseudowords (which participants typically find harder to reject). This task taps sensitivity to morpheme units in reading.
- ***Spelling (items selected from Burt & Tate, 2002)*.** Participants completed a spelling-to-dictation task comprised of 40 English words (see also Ulicheva, Coltheart, et al., 2021; Ulicheva, Marelli, et al., 2021). They first heard the to-be-spelled word in isolation then in a sentence that provided contextual information, e.g., “Dissuade—His friends tried to dissuade him from flying.” Responses were typed and could be corrected using the backspace button, before pressing enter for the next word. Responses were scored as correct or incorrect and proportion correct / 40 was used for all analyses.
- ***Nonword Repetition subtest from the Comprehensive Test of Phonological Processing (CToPP-2, Wagner et al., 2013)*.** Participants listened to and repeated 30 pseudowords of increasing length and complexity. Responses were recorded and scored offline as correct/incorrect by two experienced raters (second and third authors), with any disagreements reviewed by one rater. Proportion correct / 30 was used for all analyses.
- ***Vocabulary (Shipley, 1940)*.** Participants read a word on the centre of the screen and decided which of four words written below was the most similar in meaning. There was a total of 40 items, scored as correct or incorrect, and proportion correct / 40 was used for all analyses.
- ***Spoonerisms subtest from the Phonological Assessment Battery (PhAB; Frederickson et al., 1997)*.** Participants heard two words and were instructed to repeat them aloud, but swapping the beginning (spoonerisms first, 20 items, e.g., basket-lemon > lasket-bemon) or final (spoonerisms last, 20 items, e.g., rabbit-napkin > rabbin-napkit) phonemes. Responses were recorded and scored offline as correct if both phonemes were correctly swapped and incorrect otherwise. The first and last versions were treated separately in analyses using proportion correct / 20 as the dependent measure.

### Neuroimaging Data Collection

The imaging acquisition consisted of task-based fMRI measures of reading and language processing, T1-weighted anatomical scans, and the resting-state sequence that is the focus of this manuscript. Diffusion-weighted structural data were also acquired for the majority of the participants. MRI data were collected using a 3T Siemens Trio scanner, with a 32-channel head coil. High resolution T1-weighted anatomical images were acquired using a magnetization prepared rapid acquisition gradient echo (MPRAGE) protocol (TR = 2,250 ms, TE = 2.99 msec, flip angle = 9°, 1 mm thick slices, 256 × 240 × 192 matrix, voxel size: 1 × 1 × 1 mm). Whole-brain resting-state fMRI data were acquired using an echoplanar imaging (EPI) sequence based on the Siemens EP2D sequence (duration = 10 min, TR = 2,000 ms, TE = 3 ms, flip angle = 78°, 3 mm thick slices, 64 × 64 × 32 × 300 matrix, voxels size: 3 × 3 × 3.75 mm).

### Neuroimaging Analysis Pipeline

The resting-state data were converted from native DICOM format to NifTI-1 format using the dcm2nii conversion tool (RRID: SCR_014099). Subsequently, the resting-state fMRI data and T1-weighted anatomical images were processed using Ciftify (Dickie et al., 2019), which employs fMRIPrep Version 1.3.2 (Esteban et al., 2019; RRID:SCR_016216) for preprocessing. The following text describes the processing steps:

Preprocessing was performed using fMRIPrep, a Nipype (Gorgolewski et al., 2011; RRID:SCR_002502) based tool. Each T1-weighted (T1w) volume was corrected for intensity non-uniformity using N4BiasFieldCorrection Version 2.1.0 (Tustison et al., 2010) and skull-stripped using antsBrainExtraction.sh Version 2.1.0 (Cook, 2020; using the OASIS template). Brain surfaces were reconstructed using recon-all from FreeSurfer Version 6.0.1 (Fischl et al., 1999; RRID:SCR_001847), and the brain mask estimated previously was refined with a custom variation of the method, to reconcile Advanced Normalization Tools (ANTs)-derived and FreeSurfer-derived segmentations of the cortical grey matter (GM) of Mindboggle (Klein et al., 2017; RRID:SCR_002438). Following best practice recommendations, the surface reconstructions were visually inspected for errors in skull-stripping and surface topography. These errors were manually corrected and the surface reconstruction was re-started from the appropriate step in the FreeSurfer recon-all pipeline. Spatial normalisation to the ICBM 152 Nonlinear Asymmetrical template version 2009c (Fonov et al., 2011; RRID:SCR_008796) was performed through nonlinear registration with the antsRegistration tool of ANTs Version 2.1.0 (Avants et al., 2008; RRID:SCR_004757), using brain-extracted versions of both T1w volume and template. Brain tissue segmentation of cerebrospinal fluid (CSF), WM, and GM was performed on the brain-extracted T1w using the FMRIB's Automated Segmentation Tool (FAST; Zhang et al., 2001; FSL Version 5.0.9, RRID:SCR_002823).

Functional data were slice time corrected using *3dTshift* from AFNI Version 16.2.07 (Cox, 1996; RRID:SCR_005927) and motion corrected using *mcflirt* (Jenkinson et al., 2002; FSL). This was followed by co-registration to the corresponding T1w using boundary-based registration (Greve & Fischl, 2009) with six degrees of freedom, using FreeSurfer's *bbregister*. Motion correcting transformations, BOLD-to-T1w transformation and T1w-to-template (Montreal Neurological Institute [MNI]) warp were concatenated and applied in a single step using *antsApplyTransforms* using Lanczos interpolation. Frame-wise displacement (Power et al., 2014) was calculated for each functional run using the implementation of Nipype. To correct for the effects of movement, we regressed nuisance signals calculated through *fMRIPrep*, namely, translations and rotations in three dimensions, their temporal derivative, squared term, derivative of the squared term, and signals within CSF and WM masks (Satterthwaite et al., 2012).

Following processing through fMRIPrep, seed-based functional connectivity of the VWFA with the rest of the cortex in the left hemisphere was calculated using Connectome Workbench (Version 1.5.0; WUSM Human Connectome Project, 2024) functions. For seed-based correlation analysis, the MNI coordinates for the VWFA (−42, −57, −15; Cohen et al., 2002) were projected to the nearest vertex on the mid-thickness surface of the left hemisphere for each participant. Subsequently, a circular ROI was created around this vertex. Following previous reports, our main analyses focused on a sphere with 10 mm diameter but we also tested if similar results were observed with 5 and 8 mm. Next, the correlation of the mean signal within the ROI was calculated across the whole left hemisphere. We transformed the Pearson correlation coefficients to *z*-scores using Fisher’s *r*-to-*z* transform. The map of VWFA *z*-transformed correlations for each vertex of the left hemisphere surface constitutes the measured variable for the resting-state fMRI analysis. We used cluster-free threshold enhancement with 10,000 repetitions as implemented in FSL's PALM tool for the statistical analysis of associations between behavioural measures and VWFA connectivity (Smith & Nichols, 2009).

### Meta-Analytic Regions-of-Interest Analysis

For further region of interest (ROI)-based analysis, we focused on regions identified by Taylor et al. (2013). We selected ROIs in the anterior fusiform gyrus, dIFG, IPC, and MTG that were associated with phonological or lexical-semantic processes in the meta-analysis. We used the original clusters identified in the meta-analysis and projected them to the cortical surface using Connectome Workbench functions.

### Network-Based Statistics

We performed an additional analysis probing the link between behavioural metrics and resting-state brain networks using network-based statistics (NBS; Zalesky et al., 2010). Unlike mass-univariate analysis, which evaluates network edges individually, NBS assesses the entire network structure, enhancing statistical power. It identifies clusters of interconnected edges with significant associations to behavioural measures. The method ensures the reliability of results by comparing the actual network to a null model generated through permutation testing. By focusing on interconnected edges, NBS can be more sensitive to subtle but widespread changes in connectivity patterns, potentially offering greater sensitivity than methods analysing isolated connections. For NBS, we calculated the Pearson correlation between ROI timeseries in the 100-region parcellation provided by Schaefer et al. (2018). The association between resting-state connectivity and behavioural performance was analysed using Network-Based R-Statistics for Mixed-Effect Models toolbox Version 0.1.5 (Gracia-Tabuenca, 2022). The significance criterion for significant clusters was adjusted for multiple comparisons across measures (*p* < 0.00625 for eight measures).

## RESULTS

### Correlations Among Behavioural Measures

**Figure 2.**
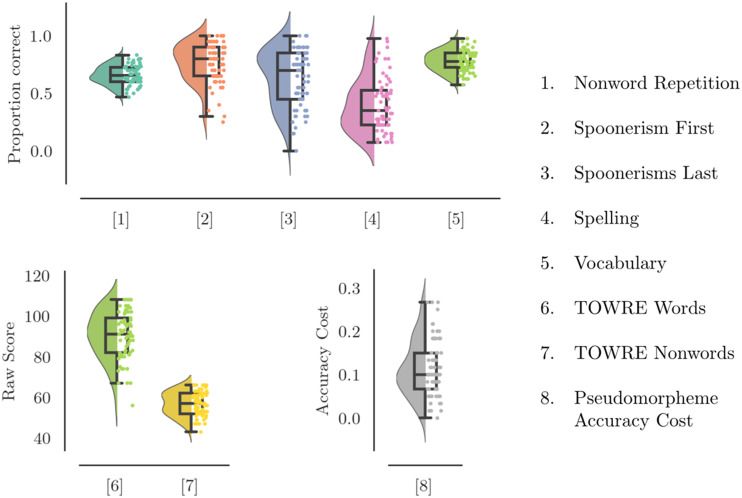
Performance on behavioural tasks. The black outline represents a boxplot with whiskers from the 5th to the 95th percentile, a box from the 25th to the 75th percentile, and a line at the median. The shaded area in the background of the figure shows the kernel density estimation for a normal distribution fitted to the observed data (violin plot). The dots on the right half of each figure show the individual data points with a random jitter to avoid overlap.

Figure 2 shows boxplots of performance on all behavioural tasks and Table 1 shows correlations among the behavioural measures. There were positive correlations between nonword reading, nonword repetition, spoonerisms, and spelling, all of which involve phonological processing. Spelling also positively correlated with vocabulary, consistent with the idea that this task also indexes lexical-semantic knowledge. However, the vocabulary and pseudomorpheme lexical decision tasks were both expected to tap lexical-semantic processes but were uncorrelated. In addition, sight word reading efficiency and spelling were both expected to involve phonological and lexical-semantic processes, but, again, were uncorrelated.

**Table 1.** Correlations and mean/variation of scores among behavioural measures

Variable	*M*	*SD*	1	2	3	4	5	6	7
1. Nonword Repetition	0.66	0.08							
2. Spoonerism First	0.76	0.17	.34** [.12, .54]						
3. Spoonerism Last	0.63	0.25	.27* [.03, .47]	.53** [.34, .68]					
4. Vocabulary	0.78	0.09	.09 [−.15, .32]	.27* [.04, .48]	.26* [.02, .47]				
5. Spelling	0.40	0.23	.43** [.22, .61]	.50** [.30, .66]	.36** [.14, .55]	.60** [.42, .73]			
6. TOWRE Word	90.39	11.67	−.04 [−.27, .20]	.00 [−.23, .24]	.02 [−.22, .26]	.00 [−.23, .24]	−.06 [−.29, .18]		
7. TOWRE Nonword	56.80	5.85	.29* [.06, .50]	.25* [.01, .46]	.21 [−.03, .47]	.16 [−.08, .38]	.43** [.21, .60]	.44** [.22, .61]	
8. Pseudomorpheme Accuracy Cost	0.12	0.06	−.13 [−.36, .11]	−.14 [−.37, .10]	−.26* [−.47, −.03]	−.09 [−.32, .15]	−.06 [−.29, .18]	−.09 [−.32, .15]	−.20 [−.42, .03]

*Note*. *M* and *SD* are used to represent mean and standard deviation, respectively. Values in square brackets indicate the 95% confidence interval for each correlation. The confidence interval is a plausible range of population correlations that could have caused the sample correlation (Cumming, 2014). * indicates *p* < .05 ** indicates *p* < .01.

### Whole Left-Hemisphere RSFC With the VWFA Seed

**Figure 3.**
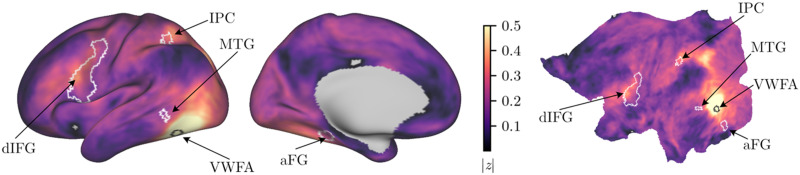
Surface correlation analysis with highlighted regions of interest along the dorsal and ventral pathway. The left panel shows a lateral and medial view of the inflated left surface. The right panel shows a flat map of the left hemisphere. The white outlines show key dorsal and ventral pathway clusters that showed [pseudoword–word] or [word–pseudoword] activation differences in Taylor et al. (2013). The values represent correlation values after Fisher *r*-to-*z* transformation. All correlations were significant at *p*_TFCE_ < 0.05, except for small regions in the inferior frontal gyrus and medial cingulate gyrus shown in grey. The surface visualizations are based on the s12000 average surface of the Human Connectome Project.

As an initial step, we evaluated the correlation between a VWFA seed with the rest of the brain. The results of this analysis are shown in Figure 3 and Table 2. Figure 3 and Table 3 also show the correspondence between this seed correlation map and clusters in key dorsal and ventral pathway regions that are proposed to be involved in print-to-sound and print-to-meaning mapping, respectively (Taylor et al., 2013). These clusters are projected onto the cortical surface, and it can be seen that there are relatively high correlations between the VWFA seed and regions that overlap with these clusters. Table 2 also reveals correlations with the left insula, STG, and precuneus.

**Table 2.** Peak correlations of the visual word form area seed with the rest of the left cortex

#	Peak value	DK	MMP	Yeo7
1	1.0	fusiform (17.7%)	L_FCC (4.4%)	3 (35.6%)
2	0.47	superior parietal (19.4%)	L_IPS1 (2.7%)	3 (35.6)
3	0.38	pars opercularis (30%)	L_IFJa (12.5%)	3 (26%)
4	0.30	insula (100%)	L_FOP2 (44.4%)	4 (76.9%)
5	0.29	precentral (100%)	L_6d (58.6%)	2 (96%)
6	0.27	superior temporal (100%)	L_A4 (77.4%)	2 (100%)
7	0.27	precuneus (100%)	L_V2 (100%)	1 (100%)

*Note*. Peak values indicate the Pearson correlation coefficient. Only correlations above 0.25 are shown. A minimum peak distance of 20 mm was used. The other columns show the percentage of overlap with regions in the Desikan-Killiany (DK), Multimodal Parcellation (MMP), and Yeo 7-Network atlas.

**Table 3.** Mean correlation values derived from the VWFA correlation map for regions of interest as identified in Taylor et al. (2013)

ROI	Pearson correlation		
	Mean	SE	Percentile rank
Intraparietal cortex	0.31	0.025	95.2%
Anterior fusiform gyrus	0.24	0.019	85.9%
Dorsal inferior frontal gyrus	0.24	0.016	85.6%
Middle temporal gyrus	0.20	0.020	69.4%

*Note*. As a reference, the percentile rank of the correlation strength compared to all regions in the Multimodel Parcellation (MMP) atlas is shown.

### Correlations Between Behavioural Measures and Whole Left Hemisphere RSFC With the VWFA Seed

**Figure 4.**
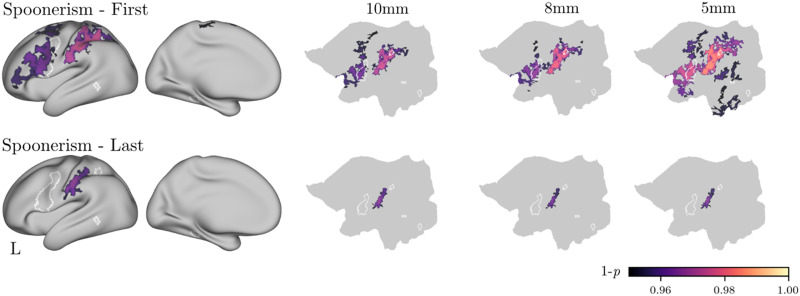
Association between phonological measures (Spoonerism First, Spoonerism Last) and resting state functional connectivity (RSFC) of the visual word form area in the whole-brain analyses. The white outlines show ROIs identified by Taylor et al. (2013). The areas filled with colour indicate significant clusters after TFCE correction. The three-dimensional visualizations on the left show the results with a 10 mm seed sphere. The flat maps on the right show the results at different seed radii.

We correlated the map of the VWFA *z*-transformed correlations for each vertex of the left hemisphere surface with each individual behavioural measure and corrected for multiple comparisons using threshold-free cluster enhancement (TFCE) with 10,000 permutations. Figure 4 shows the results of these analyses.

- **Phonological measures**. Spoonerisms (first and last) task performance was positively correlated with RSFC between the VWFA and dIFG, PCG, and superior and intraparietal cortex, which are dorsal stream regions (see Table 4 for peak coordinates). This result held for all three VWFA seed radii. Nonword reading and nonword repetition performance did not correlate with VWFA connectivity with any brain regions in the whole brain anaylsis.
- **Lexical-semantic measures**. Neither vocabulary score nor morpheme sensitivity were correlated with VWFA connectivity in the whole-brain analyses.
- **General reading/spelling measures**. Neither spelling accuracy nor sight word reading efficiency were correlated with VWFA connectivity in the whole-brain analysis.

**Table 4.** Description of peak coordinates for clusters identified as showing significant associations between spoonerisms first (SpF) and last (SpL) and RSFC with the visual word form area in whole-brain analyses

Measure	#	Area	DK	MMP	Yeo7
SpF	1	173976	L_paracentral (89.8%)	L_4 (83.0%)	7 (22.7%)
			L_precentral (10.2%)	L_5m (11.7%)	1 (19.0%)
					2 (14.9%)
					6 (12.7%)
	2	168	L_superiorfrontal (52.8%)	L_6a (40.4%)	2 (100.0%)
			L_caudalmiddlefrontal (24.6%)	L_FEF (20.5%)	
			L_precentral (22.5%)	L_6ma (19.9%	
	3	1417	L_superiorparietal (32.7%)	L_2 (16.6%)	3 (52.3%)
			L_postcentral (30.9%)	L_AIP (14.5%)	4 (17.3%)
			L_supramarginal (26.4%)		6 (14.2%)
			L_inferiorparietal (10.0%)		2 (12.5%)
	4	4559	L_parsopercularis (27.7%)	L_6r (12.8%)	6 (51.1%)
			L_precentral (21.2%)	L_8C (11.2%)	7 (22.5%)
			L_rostralmiddlefrontal (18.8%)	L_p47r (10.4%)	3 (15.7%)
			L_parstriangularis (16.5%)	L_45 (10.2%)	
			L_caudalmiddlefrontal (14.3%)		
SpL	1	1217	L_postcentral (56.8%)	L_2 (33.0%)	2 (45.8%)
			L_supramarginal (40.6%)	L_PFt (23.0%)	3 (35.8%)
				L_PFop (17.4%)	4 (18.4%)

*Note*. The areas with >10% overlap in the Desikan-Killiany (DK), Multimodal Parcellation atlas (MMP), and Yeo 7-Network atlas are presented with the percentage of overlap in brackets.

### ROI Analyses

An exploratory analysis examined whether there were associations between behavioural measures of reading and VWFA functional connectivity within ROIs in left hemisphere dorsal and ventral pathway clusters documented in the preregistration. For this analysis, the statistical comparison was restricted to binary masks of the ROIs (see white outlines in Figure 3), which were based on the meta-analytic maps reported by Taylor et al. (2013). The aim of this analysis was to identify localised associations between the behavioural measures and VWFA functional connectivity within the relatively large meta-analytic ROIs.

#### Phonological measures

**Figure 5.**
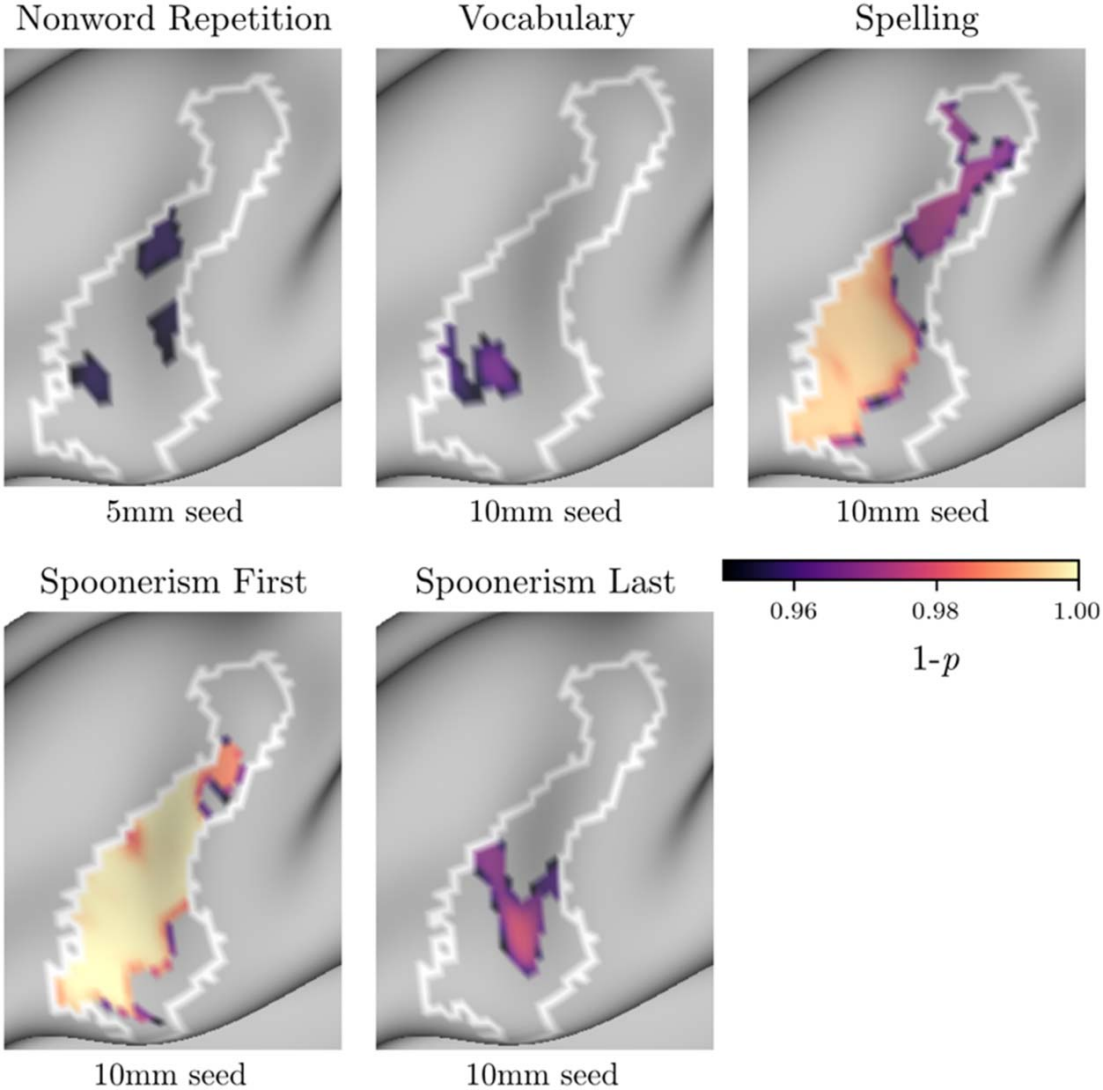
Results of the ROI-based analysis. RSFC between the VWFA and the dIFG ROI was positively correlated with performance on nonword repetition, spelling, spoonerisms (first and last), and vocabulary, at different VWFA seed sizes.

Mirroring the whole-brain analyses, spoonerisms (first and last) task performance was associated with RSFC between the VWFA and the dIFG ROI across all seed sizes (Figure 5). Nonword repetition performance also correlated with RSFC between the VWFA and the dIFG ROI, but only when using a 5 mm sphere.

#### Lexical-semantic measures

There was an association between vocabulary score and VWFA RSFC with the dIFG ROI, but only when using 8 or 10 mm VWFA seed spheres. There was no correlation between morpheme sensitivity and VWFA RSFC with any ROIs.

#### General reading/spelling measures

There was an association between spelling accuracy and VWFA connectivity with the dIFG ROI at all VWFA seed sizes. Sight word-reading efficiency did not correlate with VWFA RSFC with any of the ROIs.

### Network Based Statistics: Preregistered Exploratory Analysis

**Figure 6.**
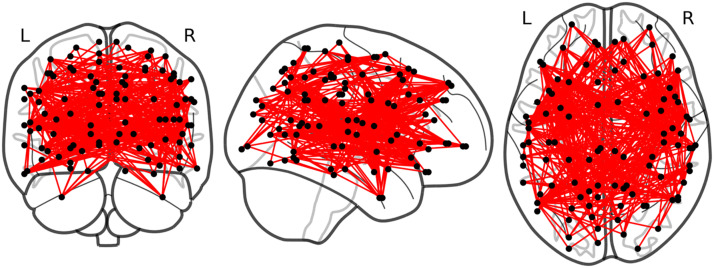
Visualization of the network based statistics result. Black dots indicate nodes and red lines indicate edges in the cluster that were significantly associated with vocabulary performance.

We conducted an additional analysis to test the association between behavioural measures and resting-state brain architecture using network-based statistics (Zalesky et al., 2010). This method examines associations between the behavioural measures and the correlation between any brain areas, that is, not just with the VWFA. Figure 6 shows that there is an association between a very large cluster and vocabulary—people who have better connectivity in that cluster perform better on the vocabulary task. The cluster is so big that it does not provide any mechanistic insight. It potentially reflects the fact that vocabulary can be used as a measure of general ability.

## DISCUSSION

Our study examined which brain areas show temporally correlated neural activity with the VWFA at rest, and how these temporal correlations relate to reading and related skills. We analysed RSFC data from 69 typical adults who also participated in a number of different behavioural tasks thought to tap phonological, lexical-semantic, or general reading/spelling processes. We first established that the VWFA seed ROI showed significant whole-brain correlations with several left hemisphere brain regions known to be involved in translating print-to-sound (dorsal IFG, IPC) or print-to-meaning (aFG, MTG) during reading (Taylor et al., 2013). Correlations with dorsal stream regions align with previous findings with native English speaking adults (Cheema et al., 2021; Koyama et al., 2010; Stevens et al., 2017), whereas those with ventral stream regions have not been prominent in previous work.

We next examined the relationship between VWFA RSFC and tasks thought to tap specific reading subprocesses. Our first hypothesis was that performance on phonological tasks (nonword reading, nonword repetition, spoonerisms) would be positively correlated with RSFC between the VWFA seed and dorsal stream regions, specifically left IPC and dIFG. The results provided some support for this hypothesis. Whole-brain analyses demonstrated that performance on the spoonerisms tasks (first and last) positively corelated with RSFC between VWFA and left dIFG, PCG, and superior and intraparietal cortex. ROI analyses supported the dIFG findings for the spoonerisms tasks, and also showed that RSFC between the VWFA and dIFG was positively correlated with performance on the nonword repetition task (though only when using a 5 mm sphere for the VWFA seed). Spoonerisms is a complex task that requires phonological memory and manipulation. The strong relationship between VWFA-dIFG RSFC and spoonerisms, as well as the weaker relationship with nonword repetition, provides converging evidence that dIFG is involved in phonological output processes.

The spoonerisms task also likely draws on sound-to-print and print-to-sound conversion since visualising the written forms of the two words (e.g., basket-lemon), may assist with the task. The whole-brain correlations observed between this task and RSFC between VWFA and left IPC are therefore consistent with assertions that this region plays a role in mapping between not only print and sound (Taylor et al., 2013, 2014), but also the spatial attention aspects of reading (Vogel et al., 2012). It is, however, surprising that similar relationships were not observed between VWFA and dorsal stream RSFC and nonword reading, which should be the most direct index of print-to-sound mapping processes. However, though performance on all three phonological tasks correlated with each other, these correlations were relatively small ∼*r* = 0.23, and nonword reading had a somewhat restricted distribution of performance relative to nonword repetition and spoonerisms.

Our second hypothesis was that performance on lexical-semantic tasks (vocabulary, sensitivity to morpheme units in reading) would be positively correlated with RSFC between the VWFA seed and ventral regions, specifically aFG and MTG. We found no evidence to support this hypothesis—accuracy cost on the pseudomorpheme lexical decision task was not correlated with RSFC between VWFA and any brain regions and vocabulary score was only correlated with VWFA-dIFG RSFC (with 8 or 10 mm VWFA seed spheres in the exploratory ROI analysis). The vocabulary task involved choosing which of four written words was a synonym for a target word, and we expected the primary demands to be on semantic selection processes. It is therefore surprising that the only relationship we observed was with RSFC between the VWFA and a region thought to be involved in phonological output processes. We also observed that performance on the vocabulary and spoonerisms tasks was correlated. Together these results suggest that the former did not specifically isolate lexical-semantic processes and involved some form of phonological processing. Furthermore, the vocabulary task may also have tapped into more general cognitive abilities, which is supported by the results of our preregistered network-based analysis in which RSFC within a very large cluster was positively correlated with vocabulary. Overall, this vocabulary task may not be an ideal measure for examining the relationship between reading related skills and RSFC since it likely draws on many different cognitive and linguistic processes.

The lack of correlation between VWFA and ventral stream RSFC and the morpheme sensitivity task was more surprising. This task involved making lexical decisions to words and pseudowords, with half the pseudowords comprising real morphemes (e.g., “towerly,” “earist”). These are hard to reject if participants are sensitive to their morphological composition and the morphemic sensitivity measure is the accuracy for standard pseudowords minus the accuracy for pseudomorphemic pseudowords. Yablonski et al. (2019) analysed diffusion tensor imaging data from a subset of the participants (*N* = 45) who took part in the current study and reported that morphemic sensitivity correlated with diffusivity measures within ventral stream tracts, namely the inferior frontal-occipital fasciculus and the inferior longitudinal fasiculus. Functional imaging data also support the idea that morphemic processing during visual word recognition depends on ventral stream regions as reviewed in Rastle (2019). The reason for the null effect observed in the current study is not clear. It could be that the morpheme sensitivity task did not capture stable individual differences that correlate with resting brain activity patterns, though this would be surprising given the relationships observed with DTI measures in Yablonksi et al.. Another possibility is that the role of brain dynamics in morpheme processing might not be adequately estimated by average resting-state activity. Further research is necessary to fully understand how morpheme sensitivity is related to functional and structural properties of the ventral pathway.

Our final hypothesis was that sight word reading efficiency and spelling performance would be positively correlated with connectivity between the VWFA seed and both dorsal and ventral regions, since these tasks make demands on both phonological and lexical-semantic processes. We found some support for this hypothesis—the exploratory ROI analyses indicated that spelling score correlated with RSFC between VWFA and dIFG at all VWFA seed sizes. However, spelling did not correlate with RSFC between VWFA and ventral stream regions, and sight word reading efficiency was not associated with RSFC between VWFA and any region. The idea that spelling draws on both lexical-semantic and phonological skills is supported by the medium-to-strong correlations with vocabulary, nonword repetition, spoonerisms, and nonword reading. Functional imaging and neuropsychological data also support this assertion, since spelling skills are associated with activity in/damage to both dorsal and ventral stream regions (Taylor & Rapcsak, 2023). Our RSFC data therefore reflect the phonological but not the lexical-semantic demands of the spelling task. This may be because we used spelling-to-dictation rather than, for example, writing a word to match a written definition, though participants did hear the word both in isolation and in a sentence. The lack of a relationship between VWFA RSFC and sight word reading efficiency is notable given that whole-word reading tasks are the most commonly used in RSFC studies examining reading networks. However, two studies that used sight word efficiency from the TOWRE (Cheema et al., 2021; Stevens et al., 2017) also reported no significant correlations between this measure and left fusiform RSFC, and another reported a significant association only between left fusiform and thalamus RSFC (Cross et al., 2021). Thus, our results are not inconsistent with previous findings. Furthermore, in our sample of typical adults, sight word reading efficiency did not correlate with any other behavioural measures except nonword reading efficiency, which also showed no association with VWFA RSFC. It is possible that these speeded measures largely tap general processing speed rather than particular reading skills, within the population studied here.

## SUMMARY AND LIMITATIONS

We conducted a well-powered study with 69 typical native English speaking adults and preregistered analyses to examine the relationship between VWFA resting state functional connectivity and tasks that tapped multiple reading and related skills. VWFA activity was temporally correlated with activity in both dorsal (dIFG, IPC) and ventral (aFG, MTG) reading regions. Correlations with behavioural performance revealed associations with the former but not the latter. Specifically, high ability in tasks that tap phonological processing and/or print-to-sound mapping (spoonerisms, spelling, and, less reliably, nonword repetition) was associated with stronger connectivity between the VWFA and dIFG; spoonerisms performance was also associated with RSFC between VWFA and IPC. Conversely, no associations with behavioural performance were observed for RSFC between VWFA and ventral brain regions.

It may be that the most robust associations were observed with spoonerisms and spelling because these tasks are complex and performance was highly variable between participants. As already discussed, these tasks draw on multiple subprocesses, including verbal working memory, mapping between print and sound (and vice versa), and phonological manipulation, with spelling additionally calling on lexical-semantic knowledge. They perhaps therefore provide greater opportunity for observing resting-state brain behaviour associations than our other measures. However, the complexity of these tasks is also a limitation since it is difficult to draw firm conclusions about what specific subprocess drives the associations. One approach for the future would be to construct latent variables from multiple indicators expected to load on the same underlying reading subprocess, which would minimise the noise in the behavioural data, an approach that is common in developmental studies (e.g., Cunningham et al., 2021). This approach might enhance the opportunity to observe the predicted distinction between processes associated with connectivity between the VWFA and dorsal versus ventral regions.

## ACKNOWLEDGMENTS

Special thanks to Clare Lally for her help collecting the data analysed in this manuscript.

## FUNDING INFORMATION

Kathleen Rastle, Economic and Social Research Council (https://dx.doi.org/10.13039/501100000269), Award ID: ES/L002264/1.

## AUTHOR CONTRIBUTIONS

**Joe Bathelt**: Conceptualization; Data curation; Formal analysis; Methodology; Software; Validation; Visualization; Writing – original draft; Writing – review & editing. **Kathleen Rastle**: Conceptualization; Funding acquisition; Investigation; Methodology; Writing – review & editing. **J. S. H. Taylor**: Conceptualization; Data curation; Formal analysis; Investigation; Methodology; Project administration; Supervision; Validation; Writing – original draft; Writing – review & editing.

## DATA AVAILABILITY STATEMENT

The analyses reported in this manuscript were preregistered (https://osf.io/qfc4u), and all data, scripts and experimental materials are available for peer review on the Open Science Framework (https://osf.io/yhf2e/) and neuroimaging data are available on OpenNeuro (https://doi.org/10.18112/openneuro.ds004765.v1.0.0).

## TECHNICAL TERMS

Intraparietal cortex (IPC): Dorsal stream region involved in mapping print to sound during reading.
Dorsal inferior frontal gyrus (dIFG): Frontal lobe area involved in speech and phonological output.
Anterior fusiform gyrus (aFG): Ventral stream region for visual processing, word recognition, and semantics.
Middle temporal gyrus (MTG): Brain region involved in semantic processing.
Resting-state functional connectivity (RSFC): Measures brain region correlations during non-task states using fMRI.
Visual word form area (VWFA): Brain area in left occipito-temporal cortex, specialises in word and letter recognition.
Phonological tasks: Tasks that repeat/manipulate sounds, translate print to sound.
Dorsal pathway: Includes intraparietal cortex and dorsal inferior frontal gyrus and involved in translating print to sound.
Ventral pathway: Includes aFG and MTG and involved in translating print to meaning.
